# Identification of haplotype tag single nucleotide polymorphisms within the nuclear factor-κB family genes and their clinical relevance in patients with major trauma

**DOI:** 10.1186/s13054-015-0836-6

**Published:** 2015-03-20

**Authors:** Wei Pan, An Qiang Zhang, Wei Gu, Jun Wei Gao, Ding Yuan Du, Lian Yang Zhang, Ling Zeng, Juan Du, Hai Yan Wang, Jian Xin Jiang

**Affiliations:** State Key Laboratory of Trauma, Burns and Combined Injury, Institute of Surgery Research, Daping Hospital, Third Military Medical University, Gaotanyan Street, Chongqing, 400038 China; Chongqing Emergency Medical Center, Jiankang Road, Chongqing, 400042 China

## Abstract

**Introduction:**

Nuclear factor-κB (NF-κB) family plays an important role in the development of sepsis in critically ill patients. Although several single nucleotide polymorphisms (SNPs) have been identified in the NF-κB family genes, only a few SNPs have been studied.

**Methods:**

A total of 753 patients with major blunt trauma were included in this study. Tag SNPs (tSNPs) were selected from the NF-κB family genes (*NFKB1, NFKB2, RELA, RELB and REL*) through construction of haplotype blocks. The SNPs selected from genes within the canonical NF-κB pathway (including *NFKB1, RELA and REL*), which played a critical role in innate immune responses were genotyped using pyrosequencing method and analyzed in relation to the risk of development of sepsis and multiple organ dysfunction (MOD) syndrome. Moreover, the rs842647 polymorphism was analyzed in relation to tumor necrosis factor α (TNF-α) production by peripheral blood leukocytes in response to bacterial lipoprotein stimulation.

**Results:**

Eight SNPs (rs28362491, rs3774932, rs4648068, rs7119750, rs4803789, rs12609547, rs1560725 and rs842647) were selected from the NF-κB family genes. All of them were shown to be high-frequency SNPs in this study cohort. Four SNPs (rs28362491, rs4648068, rs7119750 and rs842647) within the canonical NF-κB pathway were genotyped, and rs842647 was associated with sepsis morbidity rate and MOD scores. An association was also observed between the rs842647 A allele and lower TNF-α production.

**Conclusions:**

rs842647 polymorphism might be used as relevant risk estimate for the development of sepsis and MOD syndrome in patients with major trauma.

**Electronic supplementary material:**

The online version of this article (doi:10.1186/s13054-015-0836-6) contains supplementary material, which is available to authorized users.

## Introduction

Trauma is a major public health problem worldwide, ranking as the third leading cause of death. With great improvements in the emergency care system, survival after major trauma is common, but it is often complicated with sepsis and multiple organ dysfunction syndrome (MODS) [1]. Therefore, preventing sepsis and MODS is crucial in the treatment of patients who survive major trauma. It has been demonstrated that inappropriate immune inflammatory response contributes to the development of sepsis and MODS in major trauma patients [2]. Increasing evidence suggests that genetic variants, particularly single nucleotide polymorphisms (SNPs), are critical determinants for inter-individual differences in both inflammatory responses and clinical outcome in sepsis patients [3,4]. Delineating the variation in genes and associated differences in response to trauma might assist in the risk stratification of trauma patients at the early stage of trauma in relation to the development of posttraumatic sepsis and MODS.

NF-κB family regulates genes required for both the innate and adaptive immune responses [5,6]. The human NF-κB family contains five members: p50, p52, p65 (RelA), RelB, and c-Rel, which are encoded by *NFKB1 [GenBank: NC_000004], NFKB2 [GenBank: NC_000010], RELA [GenBank: NG_029971], RELB [GenBank: NC_000019] and REL [GenBank: NC_000002]* genes, respectively. Recent studies have unraveled the complexity of NF-κB activation by identifying two parallel activation pathways: the canonical and non-canonical pathways [7]. The canonical pathway mainly activates the transcription of many proinflammatory cytokine and chemokine genes that initiate and propagate innate immune responses [8]. The non-canonical pathway mainly regulates adaptive immune responses and lymphoid development. Therefore, the pivotal role of NF-κB canonical pathway in the development of sepsis makes it an interesting candidate for genetic analysis.

Growing evidence indicates that allelic variation within the NF-κB family genes may influence the magnitude of proinflammatory response, thereby affecting susceptibility to acute and chronic inflammatory diseases [9]. Up to now, the majority of studies have focused on cancer susceptibility. To date, Adamzik [10] and Schafer [11] have reported that the deletion allele of the *NFKB1* insertion/deletion (−94 ins/delATTG) polymorphism is associated with increased 30-day mortality in patients with severe sepsis and septic shock. However, little is known about the association of NF-κB family gene polymorphisms with the risk of posttraumatic sepsis and MODS.

In this study, we surveyed common SNPs (minor allele frequency >0.05) located within and around the five NF-κB family genes. We then selected four SNPs (*NFKB1* rs28362491 ins/delATTG, rs4648068 A/G, *RELA* rs7119750 C/T, and *REL* rs842647 G/A) from the canonical pathway of NF-κB and investigated their clinical relevance in relation to the development of sepsis and MODS.

## Materials and methods

### Study population

A total of 753 unrelated patients with major trauma were recruited in this study. All of the participants are ethnic Han Chinese and live in the Chongqing district, southwest China. The trauma patients were consecutively admitted to the Department of Trauma Surgery in the Daping Hospital and the Chongqing Emergency Medical Center between from January 1, 2005 to June 1, 2014. They were enrolled in the study if they met the following criteria: (1) age between 18 and 65 years, (2) expected injury severity score (ISS) greater than 16 and (3) probability of survival for longer than 48 hours. Patients were not eligible if they had penetrating injuries or preexisting cardiovascular, respiratory, renal, hepatic, hematologic, or immunologic diseases. ISS were calculated according to the 2005 abbreviated injury scale developed by independent evaluators [12]. All patients requiring operative intervention received standard surgical care and treatment postoperatively in the ICU. The protocol for this study was approved by the Ethical and Protocol Review Committee of the Third Military Medical University, and informed consent was obtained from the subjects or the patient’s next of kin. Patient confidentiality was preserved according to the guidelines for studies of human subjects.

### Clinical evaluation

The patients with major trauma were monitored prospectively after admission, by physicians who did not know the genotypes. A sepsis diagnosis was made if patients met all of the following criteria: clinical evidence of infection, body temperature >38.5°C or <36.5°C, and leukocyte count >10 × 10^9^/L or <4 × 10^9^/L. Infection was defined as a clinically obvious source or positive bacterial cultures. Pneumonia was diagnosed when a predominant organism was isolated from appropriately obtained sputum cultures in the setting of purulent sputum production and/or a new or changing pulmonary infiltrate on chest radiograph. Bloodstream infections were diagnosed based on isolation of a predominant organism from blood cultures obtained under sterile conditions. The criteria for urinary tract infections included the following: >10 white blood cells per high-power field on microscopic examination or isolation of >10^5^ organisms/mL urine or >10^4^ organisms and presence of symptoms. The criteria for catheter-related infections included isolation of >15 colony forming units from catheter tips cultured only in the setting of suspected infection. A wound infection was identified by drainage of purulent material from the wound. Daily physiologic and laboratory data were collected during the ICU stay and clinical events were recorded thereafter until death or hospital discharge. MOD scores were calculated as the sum of the simultaneously obtained individual organ scores on each hospital day [13]. Neurological scoring was not performed because every patient was sedated. The MOD scores and sepsis diagnoses were determined by individuals who did not know the patients’ genotypes.

### SNP selection

The full sequence of the human *NFKB1, NFKB2, RELA, RELB and REL* genes observed in the current study included 5 kb upstream of the transcription start site, all exons and introns and 5 kb downstream of the stop codon (126.0 kb, 18.0 kb, 19.3 kb, 46.7 kb and 72.7 kb total, respectively), which were pinpointed to chromosome 4, position 102501329–102617302 (*NFKB1*), chromosome 10, position 104144219–104152270 (*NFKB2*), chromosome 11, position 65653596–65662972 (*RELA*), chromosome 19, position 50196552–50233292 (*RELB*) and chromosome 2, position 60881574–60944284 (*REL*), respectively [14] (www.ncbi.nlm.nih.gov/genbank/).

Genetic variation data for the entire *NFKB1, NFKB2, RELA, RELB and REL* genes and their surrounding selected regions were obtained from the HapMap project [15] (www.hapmap.org) for 137 Han Chinese individuals from Beijing (CHB). From this database, a total of 325 SNPs (247 in *NFKB1*, 14 in *NFKB2*, 17 in *RELA*, 18 in *RELB* and 29 in *REL*) have been identified in the CHB population (Additional file 1), and 118 (91 in *NFKB1*, 3 in *NFKB2*, 7 in *RELA*, 13 in *RELB* and 4 in *REL*) were common SNPs with a minor allele frequency (MAF) more than or equal to 0.05 (Additional file 2). Haplotype blocks were constructed using Haploview, version 4.2 (Broad Institute of MIT and Harvard, Cambridge, MA, USA), a software package that provides computation of linkage disequilibrium (LD) statistics and population haplotype patterns from genotype data [16]. Haplotype blocks represent regions inherited without substantial recombination in the ancestors of the current population. The history of recombination between a pair of SNPs can be estimated with the use of the normalized measure of allelic association D’ (value of D prime between the two loci) [17]. The criterion for the selected SNPs to construct a haplotype block is that all SNPs in one region must be in strong LD with D’ of greater than 0.98 for the upper 95% confidence bound and greater than 0.7 for the lower bound. To determine the possible functionality of the haplotype tag SNPs (htSNPs) selected from the 5′-flanking region of the NF-κB family genes, online software was used to analyze the effect of these SNPs on potential transcription factor binding sites [18] (www.targetscan.org).

### Genotyping of selected SNPs

Blood specimens were collected in tripotassium ethylenediaminetetraacetic acid sterile tubes from trauma patients immediately after admission, to avoid the effect of blood transfusion. The genomic DNA was isolated from whole blood using the Wizard genomic DNA purification kit (Promega, Madison, WI, USA) according to the manufacturer’s protocol. DNA concentration in all samples was determined by ultraviolet spectrophotometry, adjusted with sterile distilled water to a 40 μg/mL concentration and stored at −80°C. Pyrosequencing was used for genotyping [19]. Genotyping was performed in a blinded fashion without knowledge of the patients’ clinical data, and approximately 10% of the samples were genotyped in duplicate to monitor genotyping quality.

### *Ex vivo* TNF-α production

A human whole-blood assay was used as described by Majetschak *et al*. [20]. In brief, aliquots of whole blood collected from the trauma patients immediately after admission were mixed 1:1 with Roswell Park Memorial Institute (RPMI) 1640 culture medium (Thermo Scientific, Beijing, China), and incubated with 100 ng/mL lipopolysaccharide (LPS) (*Escherichia coli* O26:B6; Difco Laboratories, Detroit, MI, USA) in a sample mixer at 37°C for 4 hours. After centrifugation, the supernatants were aspirated and aliquoted for storage at −80°C. TNF-α in the supernatants was assayed with a sandwich ELISA, according to the manufacturer’s instructions (Endogen, Woburn, MA, USA). The detection limits of the assay were 4 pg/mL.

### Statistical analysis

Sample size was calculated using the online Power and Sample Size software program [21] (http://biostat.mc.vanderbilt.edu/wiki/Main/PowerSampleSize). The desired power of our study was set at 80% with a significance level of 0.05 calculated using a two-sided test. We chose the log-additive inheritance model, which is the most suitable one for polygenic diseases. On the basis of our calculations using the Power and Sample Size software program, our sample (n = 753) was considered adequate to study the selected SNPs of the NF-κB family genes.

Allele frequencies for each SNP were determined by gene counting. Genotype distribution of each SNP was tested for deviation from Hardy-Weinberg equilibrium (HWE) using chi-square (*χ*^2^) analyses. The extent of pair-wise linkage disequilibrium (*r*^2^-value) between polymorphisms was determined by the Haploview version 4.2. The association between polymorphisms and MOD scores was performed using analysis of covariance testing with age, sex ratio, and injury severity to adjust for possible confounding effects. Three genetic models (allele dose, dominant, and recessive) were used. The association of genotypes with sepsis morbidity rate was determined by *χ*^2^ analysis. Odds ratios (OR) with 95% CI were calculated by multivariate logistic regression models to estimate the relative risk of sepsis. All *P*-values were two-sided and were adjusted using the Bonferroni correction for multiple testing, with *P* <0.05 used to determine statistical significance. All statistical analyses were carried out using SPSS statistical software (version18.0; SPSS Inc, Chicago, IL, USA).

## Results

### Construction of haplotype blocks and selection of SNPs

**Figure 1:**
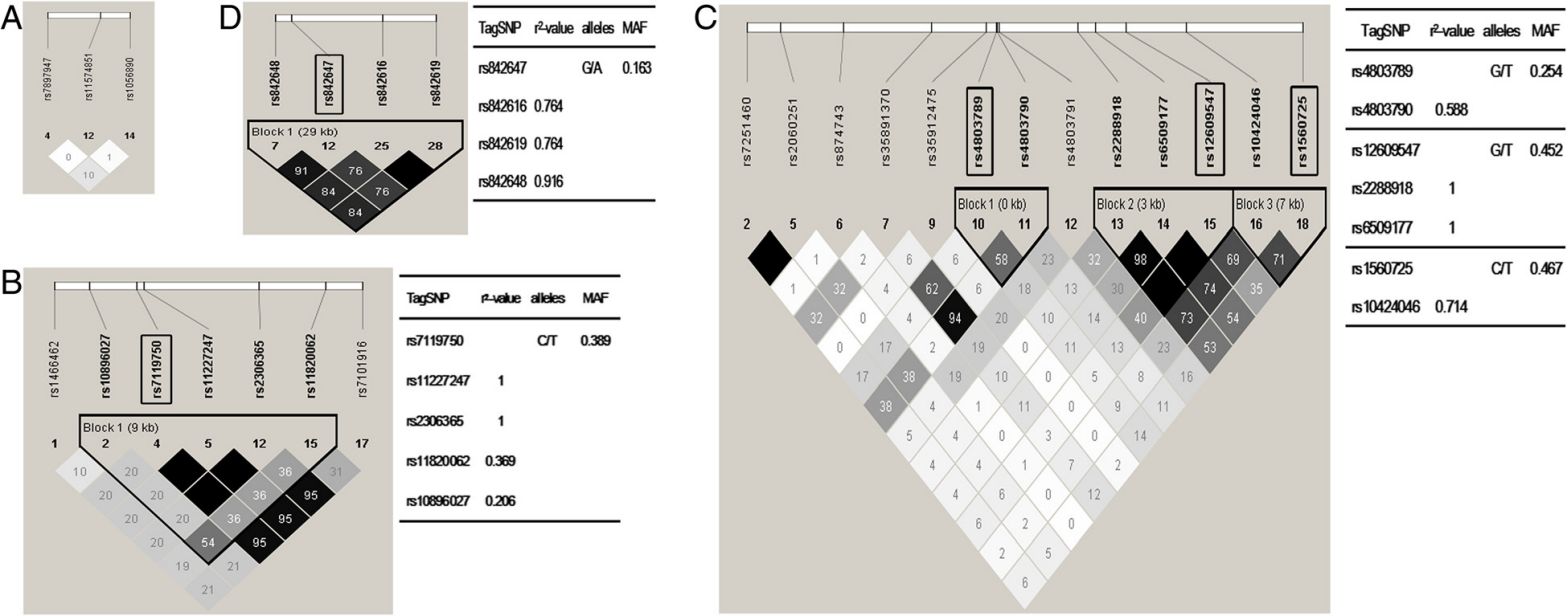
**Overview of selected haplotype tag single-nucleotide polymorphisms (htSNPs) within the entire** ***NFKB2*** **,** ***RELA*** **,** ***RELB*** **and** ***REL*** **genes.** Linkage disequilibrium (LD) plot of the SNPs with a minor allele frequency (MAF) ≥5% within the **(A)** *NFKB2*, **(B)** *RELA*, **(C)** *RELB* and **(D)** *REL* genes and 5-kb up- and downstream regions is displayed using an *r* ^2^ black and white color scheme. Black represents very high LD (*r* ^2^ = 1), and white indicates the absence of correlation (*r* ^2^ = 0) between SNPs. The three SNPs within the entire *NFKB2* gene did not have a strong correlation between each other or with others. The htSNPs and SNPs that are indirectly measured by them are listed with corresponding *r* ^2^ values. Major and minor alleles of the selected tag SNPs are given with their frequencies, on the basis of the HapMap data for Chinese individuals from Beijing.

SNPs with a MAF ≥0.05 were shown within and around the *NFKB1, NFKB2, RELA, RELB and REL* genes from the HapMap database for the CHB population (Additional file 3 and Figure 1). rs28362491, a common insertion/deletion polymorphism (−94 insertion/deletion ATTG), though it did not form any block with other SNPs, has been identified located between two putative key promoter regulatory elements in the *NFKB1* gene. The presence of ATTG deletion (allele D) resulted in the loss of binding to nuclear protein, leading to reduced promoter activity [22], thus, rs28362491 was selected.

Taken together, eight SNPs (rs28362491, rs3774932, rs4648068, rs7119750, rs4803789, rs12609547, rs1560725 and rs842647) were selected from this study (Table 1). The canonical NF-κB pathway, which mainly involves RelA/p50 and c-Rel, plays a critical role in innate immune responses [7]. Thus four SNPs (rs28362491, rs4648068, rs7119750 and rs842647) of *NFKB1, RELA and REL* genes were selected for genotyping. We did not select tSNP from *NFKB1* block1, due to it including only two SNPS (rs3774932 and 3774933) in intron regions and little was known about the role of these two polymorphisms and immune response.

**Table 1:** **Single-nucleotide polymorphisms identified within the NF-**κ**B family genes**

**Gene**	**rs number**	**Location**	**Variation**	**MAF**	**Region**
*NFKB1*	rs28362491	−94	ins/delATTG	0.447	promoter
	rs3774932	1708	G/A	0.489	intron1
	rs4648068	95928	A/G	0.407	intron14
*RELA*	rs7119750	7853	C/T	0.389	intron10
*RELB*	rs4803789	18663	G/T	0.254	intron4
	rs12609547	27303	G/T	0.452	intron7
	rs1560725	39081	C/T	0.467	3′-flanking
*REL*	rs842647	10763	G/A	0.163	intron2

Genetic variation data for the NF-κB family genes was obtained from the HapMap project for 137 members of the Chinese Han Beijing (CHB) population. MAF, minor allele frequency.

### Allele frequencies and genotype distribution of the four selected SNPs from among trauma patients

The genotyping success rates of the four selected SNPs by pyrosequencing ranged from 98.3% to 100% in our study cohort. The MAF among the 753 trauma patients was 41.8% (rs28362491), 44.7% (rs4648067), 37.6% (rs7119750) and 13.4% (rs842647), respectively, which was quite similar to those observed in the 137 unrelated individuals in the CHB cohort in the HapMap database. The genotype distribution of all four selected SNPs was in agreement with Hardy-Weinberg equilibrium (*P* >0.05) (Table 2), indicating that both allele and genotype frequencies of these selected SNPs in the population remain constant. That is to say, they are in equilibrium from generation to generation.

**Table 2:** **Distribution of the four genotyped single-nucleotide polymorphisms (SNPs) among trauma patients**

		**MAF**		**Genotypes, number (%)**			
**SNPs**	**Number**	**Patients**	**Database**	**Wild type**	**Heterozygous**	**Variant**	**HWE test**
rs28362491	753	41.8	44.7^#^	245(32.5)	386(51.3)	122(16.2)	0.16
rs4648068	752	44.7	40.7*	231(30.7)	370(49.2)	151(20.1)	0.90
rs7119750	748	37.6	38.9*	280(37.4)	374(50.0)	94(12.6)	0.07
rs842647	733	13.4	16.3*	551(75.2)	167(22.8)	15(2.0)	0.59

*Data are derived from the HapMap database for Chinese Han in Beijing (n = 137). ^#^Global MAF was derived from the NCBI database. HWE, Hardy-Weinberg equilibrium; MAF, minor allele frequency.

### Clinical association of the four selected SNPs with development of sepsis and MODS in trauma patients

The patient cohort comprised a total of 753 consecutive Han Chinese patients, 603 male and 150 female, with age 41.2 ± 13.3 years (mean ± SD) and ISS of 22.2 ± 9.5. The demographic and baseline characteristics and the clinical data of the patients are summarized in Table 3. All patients survived ≥48 hours after admission: 311 patients (41.3%) developed sepsis. Pathogens were identified as causative microorganisms in the blood cultures of 133 septic patients (42.8%). The common pathogens identified in this study cohort were *Staphylococcus aureus*, coagulase-negative staphylococci, *Klebsiella pneumoniae*, *Acinetobacter baumanii*, *Pseudomonas aeruginosa*, *Escherichia coli*, *Enterococcussp*., and *Enterobacter cloacae*. Gram-negative infections accounted for 17.0%, Gram-positive infections accounted for 11.6% and mixed infections accounted for 10.6%, respectively. The median time point for sepsis occurrence in the whole study cohort was 7 days (interquartile range, 5.0 to 9.0 days). Organ dysfunction occurred in 327 patients (43.4%) from among whom 98 (30.0%) had two or more organ dysfunctions. Among the patients with MODS, those with sepsis accounted for 72.4%. Among the patients with sepsis, the median time point for MODS occurrence was 8 days (interquartile range, 6.5 to 10.5 days). With respect to the patients without sepsis, the median time point for MODS occurrence was 5 days (interquartile range, 4.0 to 8.0 days).

**Table 3:** **Overall clinical characteristics of patients with major trauma**

**Clinical characteristics**	**Patient data (n = 753)**
Mean age ± SD, years	41.2 ± 13.3
Age range, years	18 to 65
Males/females, n	603/150
Mean ISS ± SD	22.2 ± 9.5
≥16 to <25, n	446
≥25, n	307
Injured body regions, n	
Head	392
Thorax	439
Abdomen	254
Extremities	442
Number of regions injured, n	
Two	322
Three	185
All four	43
Organ dysfunction, n (%)	327 (43.4%)
One, n	229
Two, n	68
Three or above, n	30
Sepsis, n (%)	311 (41.3%)
Source of infection (%)	
Respiratory tract infection	42.2
Primary bloodstream infection	20.2
Urinary tract infection	18.4
Catheter-associated infection	9.7
Wound infection	7.6
Others*	1.9
Pathogens (positive blood cultures), %	
Gram-negative	17.0
Gram-positive	11.6
Fungi	3.5
Mixed Gram-negative and Gram-positive	10.6
Negative blood cultures	57.2

*Other sites of infection included soft-tissue infection, bone infection and ear infection. ISS, injury severity score.

As shown in Table 4, there were no significant differences in age, sex ratio or ISS among patients stratified according to the different genotypes of each selected SNP. Among the four genetic variants selected in this study, although it was found in only 15 patients with variant homozygotes in this study cohort, only the rs842647 was shown to be significantly associated with the risk for development of sepsis and MODS in patients with major trauma. The patients carrying the variant A allele revealed a significantly lower sepsis morbidity rate and MOD scores, when compared with those carrying the G allele (*P* = 0.024 for sepsis morbidity rate and *P* = 0.013 for MOD scores in the case of a recessive effect). Data from multiple logistic regression analyses further indicated that the patients with the rs842647 polymorphism had a lower risk for developing sepsis (OR = 0.673, 95% CI = 0.532 to 0.873; *P* = 0.012) after adjusting for possible confounders, including age, sex ratio and ISS. There were no significant associations with sepsis morbidity rate and MOD scores for the other three selected SNPs (rs28362491, rs4648068 and rs7119750).

**Table 4:** **Clinical relevance of the NF-**κ**B gene polymorphisms in patients with major trauma**

**SNPs**	**Genotype**	**Number**	**Age, years**	**Sex, male/female, n**	**ISS**	**Sepsis, n (%)**	**MOD score**
rs28362491	II	245	40.5 ± 12.4	145/100	22 ± 10.3	108 (44.1)	5.0 ± 2.3
	ID	386	42 ± 13.6	306/80	21.9 ± 8.6	156 (40.4)	4.9 ± 2.1
	DD	122	40.2 ± 13.2	98/24	24 ± 10.5	47 (38.5)	4.9 ± 2.5
rs4648068	AA	231	39.6 ± 12.5	192/39	22.0 ± 10.5	98 (42.4)	4.9 ± 2.0
	AG	370	42.5 ± 13.8	294/76	22.1 ± 8.5	153 (41.4)	5.1 ± 2.4
	GG	151	40.4 ± 13.2	116/35	23.2 ± 10.3	56 (37.1)	4.5 ± 1.7
rs7119750	CC	280	40.8 ± 13.30	222/58	22.3 ± 9.9	118 (42.1)	5.0 ± 2.3
	CT	374	40.9 ± 13.2	306/68	22.5 ± 9.4	154 (41.2)	4.9 ± 2.2
	TT	94	43.9 ± 13.9	72/22	21.3 ± 8.9	38 (40.4)	5.1 ± 2.2
rs842647	GG	551	40.7 ± 13.0	438/113	22.4 ± 9.9	228 (41.4)	5.0 ± 2.3
	GA	167	42.8 ± 14.4	135/32	22.1 ± 7.9	70 (41.9)	4.8 ± 2.0
	AA	15	43.2 ± 16.6	14/1	20.5 ± 10.2	2 (13.3)	3.1 ± 1.1
*P*-value						a1	a2

ISS, injury severity score; MOD, multiple organ dysfunction; a, recessive effect (variant homozygotes versus heterozygotes + wild-type homozygotes) as analyzed by one-way analysis of variance. a1, *P* = 0.024; a2, *P* = 0.013.

### Effect of rs842647 on LPS-induced TNF-α production

**Figure 2:**
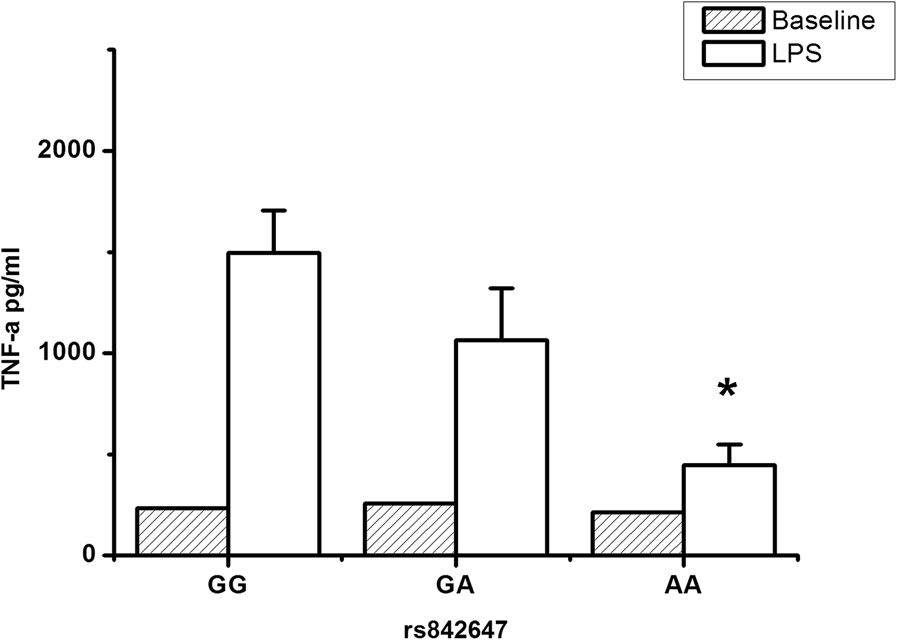
**Effect of the rs842647 polymorphism on lipolysaccharide (LPS)-induced TNF-α production.** The whole-blood samples collected from trauma patients (n = 30 for the GG and GA genotype, n = 15 for the AA genotype) immediately after admission were mixed at a 1:1 ratio (vol/vol) with RPMI 1640 culture medium and incubated with 100 ng/mL bacterial LPS at 37°C for 4 hours. TNF-α production was determined by a sandwich ELISA. The data are presented as mean ± SD. One-way analysis of variance was used to assess statistical significance. *P = 0.027 for recessive association (AA versus GG + GA).

NF-κB is a key molecule for the maturation and secretion of TNF-α [23,24]. Therefore, we hypothesized that the rs842647 polymorphism might be associated with TNF-α production. As shown in Figure 2, the rs842647 polymorphism was well associated with the LPS responsiveness of peripheral blood leukocytes. LPS-induced TNF-a production was significantly lower in patients with the variant A allele than that in those with the wild G allele (*P* = 0.027 for recessive effect) (Figure 2).

## Discussion

In the present study, to the best of our knowledge, we are the first investigators to identify the potential clinical relevance of the rs28362491, rs4648068, rs7119750 and rs842647 polymorphisms, which are identified within the *NFKB1, RELA, and REL* genes in the Han Chinese population. Tag SNPs are a subset of all variants within a chromosome region within a disease study. Use of tag SNPs is a powerful approach to investigate genetic association studies, for the genetic effect of SNPs that are not genotyped in the study can be detected through LD with a tag SNP [16]. In this case, the four SNPs selected in the current study theoretically reflect the biological significance of most genetic variations across the *NFKB1, RELA, and REL* genes because of their strong LD with other non-assayed variants.

The NF-κB, a collection of dimeric transcription factors, was first identified as a B cell factor that binds to a site in the enhancer region on the gene encoding the immunoglobulin κ light chain in 1986 [25]. The NF-κB subunits form homo- and heterodimers that control a broad spectrum of biological processes, including development, apoptosis, and the immune response [8,26]. Mice deficient in NF-κB family members exhibit defects in macrophage activation and a compromised immune response to pathogens [27]. NF-κB1 signaling is known to amplify and perpetuate inflammatory and coagulatory mechanisms prevailing in sepsis [28]. Also, NF-κB1 has also been shown to influence tissue factor in the bloodstream and, thus, activates coagulation [28-30]. In addition, the major importance of the p50 and p65 subunits in the pathophysiology of sepsis has been highlighted in both animal models and human pathology [31]. NF-κB2 is a critical mediator in the development and function of a variety of organs and cell lineages, as shown by studies of genetically modified mice in which NF-κB2 function was ablated or modified [32]. RelB is responsible for LPS-mediated attachment [33]. Moreover, c-Rel is reported to be a key NF-κB member required for host antimicrobial defenses and a regulatory transcription subunit that controls the inflammatory and immune responses in severe infection [34]. In this study, we hypothesized that common genetic variants within the canonical pathway could contribute to the development of sepsis and MODS in patients with major trauma. Four SNPs (rs28362491, rs4648068, rs7119750 and rs842647) within the canonical pathway were selected for genotyping, and they capture most of the genetic variation of *NFKB1, RELA and REL* genes and might represent the possible biological significance of their genetic variation.

Our results indicate that among the four SNPs selected in our study cohort, only the rs842647 polymorphism reveals a strong clinical relevance, with a lower rate of sepsis morbidity and MOD scores in the patients with the variant A allele. Of the other three SNPs (rs28362491, rs4648068 and rs7119750) evaluated, we did not observe a significant association. Although it has been reported to have a regulatory influence on *NFKB1* gene expression and is known to be associated with cancer [35-39], acute respiratory distress syndrome [40], periodontitis [41], autoimmune and inflammatory diseases [42], and inflammatory bowel diseases [43], the functional polymorphism rs28362491 (also known as the −94 insertion/deletion ATTG polymorphism in the promoter of *NFKB1*) was not shown to be associated with risk of sepsis and MODS in our study. Different ethnic populations and diseases, interactions or the effects of combination with non-genetic risk factors might account for these contrasting results.

Given the clinical relevance of the rs842647 polymorphism, we further hypothesized that this SNP might be associated with TNF-α production in patients with major trauma. The peripheral blood was taken immediately after admission in an attempt to avoid the potential effects of infection and fluid resuscitation. Our results reveal that the rs842647 polymorphism could affect the capacity of peripheral leukocytes to produce TNF-α, showing much lower levels of TNF-α among patients who carry the variant A allele. This is in accordance with its clinical relevance. Recently, the same variant was found to be associated to other immune-related diseases. Trynka *et al*. found that rs842647 was associated with risk of coeliac disease [44]; moreover, Amundsen *et al*. observed that coeliac disease risk polymorphisms, containing rs842647, could affect gene regulation in the thymus [45].

How might the rs842647 variant affect susceptibility to sepsis and MODS? This variant is located in the intron of the *REL* gene, which is implicated in T cell differentiation [46] and it appears to play a critical role in promoting immune and inflammatory responses [34]. A potential mechanism of the rs842647 effect is alteration of gene regulation. Online splicing prediction by SplicePort [47,48] (http://spliceport.cbcb.umd.edu/) showed that this mutation might not change gene splicing, thus, might not lead to the loss of splicing of the intron. It is possible that this variant simply amplifies the initial innate immune inflammatory response to bacterial products beyond a threshold that is tolerated by the host organ systems. Alternatively, other SNPs in high LD with this SNP may have a biological function. In the Han Chinese population the at-risk allele G is the major allele of rs842647 G/A, potentially marking a haplotype that includes many rare functional SNPs that increase risk, or the rarer A allele may actually be protective. Another, more nuanced possibility involves the modulation of regulatory T (Treg) cell function. Levels and function of Treg cells have been associated with mortality in human sepsis and have been shown to play an important role in regulating the inflammatory and antimicrobial response in animal models of sepsis [49]. Research has shown that at the Foxp3 locus encode information defining the size, the pioneer element conserved non-coding DNA sequence 3, which acts to potently increase the frequency of Treg cells generated in the thymus and the periphery, binds c-Rel in *in vitro* assays [50]. We therefore speculate that excessive *REL*–mediated stimulation of Treg cells conferred by this mutation could lead to dysregulation of Treg-cell function in patients with sepsis and lead to organ dysfunction.

Limitations of this investigation should be mentioned. First, although the sample comprised 753 trauma patients in total, the group of patients homozygous with the variant allele of the rs842647 polymorphism was relatively small (15 patients). Second, difficulties in obtaining enough blood samples from patients did not allow us to investigate the kinetics of NF-κB mRNAs levels in patients with major trauma. Therefore, the association of the four selected SNPs with changes of NF-κB mRNA expression could not be confirmed in this study. Furthermore, the patients recruited into this study cohort were limited to the Chongqing district. The generalizability of the association to populations in other regions in China is therefore not ensured. Future studies in a larger patient population might be needed to further validate their clinical relevance.

## Conclusion

In the current study, we investigated the clinical relevance of the genetic variants within the entire *NFKB1, RELA, and REL* genes by means of tag SNPs. We have demonstrated that the rs842647 polymorphism affects TNF-α production and might be used to estimate risk for sepsis and MODS in trauma patients. Further studies, both clinical and experimental, are therefore needed to confirm the significance of these findings and to investigate their synergistic effect with other genetic polymorphisms in relation to the development of sepsis in and the outcomes of trauma patients.

## Key messages

- The rs842647G allele was significantly associated with higher TNF-α production in response to *ex vivo* LPS stimulation
- The rs842647 polymorphism was closely associated with the development of sepsis and MODS
- There are no marked synergistic effects among the rs28362491, rs4648068, rs7119750 polymorphisms in relation to the development of sepsis and MODS in trauma patients

## Additional files

Additional file 1: Table S1. Single nucleotide polymorphisms identified within the NF-κB-family genes in the Chinese Han population. Additional file 2: Table S2. Single nucleotide polymorphisms with a minor allele frequency ≥0.05 in the NF-κB-family genes in the Chinese Han population. Additional file 3: Figure S1. Overview of selected haplotype tag single nucleotide polymorphisms (SNPs) within the entire *NFKB1* gene and their characteristics. (**A**) Location of the 91 SNPs within the *NFKB1* gene and 5-kb up- and downstream regions with a minor allele frequency ≥5%. The selected two haplotype tag (ht)SNPs are indicated by boxes. Linkage disequilibrium (LD) plot of the 91 SNPs in the 126.0 kb region is displayed by using *r* ^2^-black and white color scheme. Black represents very high LD (*r* ^2^ = 1), and white indicates the absence of correlation (*r* ^2^ = 0) between SNPs. (**B**) The two htSNPs and SNPs that are indirectly measured by them are listed with corresponding *r* ^2^ values. Major and minor alleles of the selected tag SNPs are given with their frequencies, on the basis of the HapMap data for the Chinese individuals from Beijing.

## Abbreviations

CHB: Chinese individuals from Beijing
ELISA: enzyme-linked immunosorbent assay
htSNP: haplotype tag single-nucleotide polymorphism
HWE: Hardy-Weinberg equilibrium
LD: linkage disequilibrium
LPS: lipopolysaccharide
MAF: minor allele frequency
MODS: multiple organ dysfunction syndrome
NF-κB: Nuclear factor-κB
OR: odds ratio(s)
PCR: polymerase chain reaction
SNP: single-nucleotide polymorphism
tSNP: tag single-nucleotide polymorphism
Treg: T regulatory
UTR: untranslated region
