# Supplementary material for: Identification of haplotype tag single nucleotide polymorphisms within the nuclear factor-κB family genes and their clinical relevance in patients with major trauma
Source: Crit Care. 2015 Mar 20;19(1):95. doi: 10.1186/s13054-015-0836-6 (PMC4404128; doi:10.1186/s13054-015-0836-6)
Supplement: Additional file 1: Table S1. — Single nucleotide polymorphisms identified within the NF-κB-family genes in the Chinese Han population. [file 13054_2015_836_MOESM1_ESM.docx]

Table S1 SNPs Identified Within the NFKB family Genes in Chinese Han Population*

| Gene | No. | Name | Position | MAF | Alleles |
| --- | --- | --- | --- | --- | --- |
| NFKB1 | 1 | rs3774932 | 103643223 | 0.489 | G:A |
|  | 2 | rs13131592 | 103645251 | 0 | A:A |
|  | 3 | rs3774933 | 103645369 | 0.419 | T:C |
|  | 4 | rs13137392 | 103645616 | 0.011 | A:G |
|  | 5 | rs3774934 | 103646506 | 0.322 | G:A |
|  | 6 | rs4380526 | 103648428 | 0 | A:A |
|  | 7 | rs4647965 | 103648469 | 0 | C:C |
|  | 8 | rs4647966 | 103648531 | 0 | A:A |
|  | 9 | rs4647968 | 103648704 | 0 | A:A |
|  | 10 | rs4647969 | 103648769 | 0 | A:A |
|  | 11 | rs4647971 | 103649086 | 0 | A:A |
|  | 12 | rs4647972 | 103649564 | 0.012 | C:T |
|  | 13 | rs4647973 | 103649617 | 0 | C:C |
|  | 14 | rs17032705 | 103652004 | 0.43 | G:A |
|  | 15 | rs961296 | 103652854 | 0 | C:C |
|  | 16 | rs3774936 | 103653167 | 0.378 | A:T |
|  | 17 | rs3774937 | 103653283 | 0.372 | T:C |
|  | 18 | rs2103487 | 103654641 | 0 | C:C |
|  | 19 | rs230537 | 103657194 | 0.012 | T:C |
|  | 20 | rs3774938 | 103657469 | 0.433 | A:G |
|  | 21 | rs4647974 | 103657904 | 0.012 | A:C |
|  | 22 | rs4647975 | 103658417 | 0 | G:G |
|  | 23 | rs4647976 | 103658807 | 0 | A:A |
|  | 24 | rs4647977 | 103659019 | 0 | G:G |
|  | 25 | rs4647978 | 103659046 | 0 | G:G |
|  | 26 | rs4235404 | 103659095 | 0.354 | C:G |
|  | 27 | rs6841533 | 103660327 | 0 | C:C |
|  | 28 | rs13132318 | 103660486 | 0 | T:T |
|  | 29 | rs13104462 | 103660509 | 0 | A:A |
|  | 30 | rs13132517 | 103660521 | 0 | T:T |
|  | 31 | rs4623003 | 103662151 | 0 | G:G |
|  | 32 | rs1599961 | 103662599 | 0.43 | G:A |
|  | 33 | rs230536 | 103662872 | 0.033 | G:A |
|  | 34 | rs1585215 | 103663504 | 0.378 | T:C |
|  | 35 | rs1585214 | 103663563 | 0.477 | C:T |
|  | 36 | rs1585213 | 103663728 | 0.43 | C:T |
|  | 37 | rs17032740 | 103663809 | 0 | A:A |
|  | 38 | rs1598856 | 103665145 | 0.488 | G:A |
|  | 39 | rs4647982 | 103665233 | 0 | G:G |
|  | 40 | rs4647984 | 103665761 | 0 | C:C |
|  | 41 | rs4647985 | 103666052 | 0 | A:A |
|  | 42 | rs4647986 | 103666198 | 0 | C:C |
|  | 43 | rs7440719 | 103666865 | 0 | G:G |
|  | 44 | rs230535 | 103667612 | 0.395 | C:A |
|  | 45 | rs170731 | 103667933 | 0.4 | A:T |
|  | 46 | rs230534 | 103668071 | 0.381 | C:T |
|  | 47 | rs7679842 | 103668362 | 0 | T:T |
|  | 48 | rs4647988 | 103668671 | 0 | C:C |
|  | 49 | rs4647989 | 103668754 | 0 | C:C |
|  | 50 | rs230533 | 103669113 | 0.4 | G:A |
|  | 51 | rs230532 | 103669197 | 0.395 | A:T |
|  | 52 | rs230531 | 103669407 | 0.395 | A:G |
|  | 53 | rs4647990 | 103670711 | 0 | G:G |
|  | 54 | rs728440 | 103671292 | 0 | G:G |
|  | 55 | rs230530 | 103673010 | 0.465 | A:G |
|  | 56 | rs4647991 | 103673481 | 0 | G:G |
|  | 57 | rs4647992 | 103674377 | 0.07 | C:T |
|  | 58 | rs4647993 | 103676405 | 0 | G:G |
|  | 59 | rs230529 | 103676448 | 0.453 | C:T |
|  | 60 | rs230528 | 103676615 | 0.453 | T:G |
|  | 61 | rs4647995 | 103676776 | 0 | G:G |
|  | 62 | rs4647996 | 103676797 | 0 | A:A |
|  | 63 | rs4647997 | 103676888 | 0 | C:C |
|  | 64 | rs230527 | 103677208 | 0 | G:G |
|  | 65 | rs7679591 | 103677448 | 0 | A:A |
|  | 66 | rs4648000 | 103677641 | 0 | A:A |
|  | 67 | rs230526 | 103677855 | 0.464 | G:A |
|  | 68 | rs230525 | 103677907 | 0.395 | A:G |
|  | 69 | rs4648002 | 103677977 | 0 | C:C |
|  | 70 | rs4648003 | 103678102 | 0 | C:C |
|  | 71 | rs230524 | 103678916 | 0 | A:A |
|  | 72 | rs4648004 | 103680136 | 0.081 | A:G |
|  | 73 | rs4648005 | 103680282 | 0 | A:A |
|  | 74 | rs4648006 | 103680588 | 0.058 | C:T |
|  | 75 | rs4648007 | 103680672 | 0 | C:C |
|  | 76 | rs4648009 | 103680849 | 0 | G:G |
|  | 77 | rs6836486 | 103681137 | 0 | T:T |
|  | 78 | rs10024951 | 103681508 | 0 | C:C |
|  | 79 | rs118882 | 103682036 | 0.405 | C:T |
|  | 80 | rs230521 | 103682357 | 0.453 | G:C |
|  | 81 | rs230520 | 103684641 | 0.395 | A:G |
|  | 82 | rs17032779 | 103685279 | 0 | T:T |
|  | 83 | rs230519 | 103685778 | 0.395 | C:T |
|  | 84 | rs17032781 | 103686108 | 0 | C:C |
|  | 85 | rs93059 | 103687547 | 0.452 | G:A |
|  | 86 | rs230516 | 103690280 | 0.4 | A:G |
|  | 87 | rs230515 | 103690463 | 0.379 | A:G |
|  | 88 | rs230514 | 103690968 | 0.395 | A:G |
|  | 89 | rs230512 | 103691920 | 0 | G:G |
|  | 90 | rs4648011 | 103694479 | 0.453 | T:G |
|  | 91 | rs4648012 | 103694607 | 0 | C:C |
|  | 92 | rs6833549 | 103694888 | 0 | C:C |
|  | 93 | rs230510 | 103695201 | 0.467 | T:A |
|  | 94 | rs230509 | 103697300 | 0.4 | A:G |
|  | 95 | rs13117745 | 103697738 | 0.081 | C:T |
|  | 96 | rs13124438 | 103698592 | 0.012 | A:G |
|  | 97 | rs230506 | 103699485 | 0 | A:A |
|  | 98 | rs230505 | 103700386 | 0.453 | T:G |
|  | 99 | rs230504 | 103700596 | 0.395 | C:T |
|  | 100 | rs230491 | 103703651 | 0.4 | C:T |
|  | 101 | rs12509950 | 103704390 | 0 | A:A |
|  | 102 | rs230492 | 103704817 | 0.398 | G:A |
|  | 103 | rs230493 | 103705253 | 0.395 | T:A |
|  | 104 | rs1598861 | 103705733 | 0.163 | A:C |
|  | 105 | rs17032815 | 103706321 | 0 | G:G |
|  | 106 | rs230495 | 103706336 | 0.465 | G:A |
|  | 107 | rs2293970 | 103707018 | 0.057 | A:T |
|  | 108 | rs230496 | 103707527 | 0.476 | A:G |
|  | 109 | rs4648013 | 103707813 | 0.024 | G:C |
|  | 110 | rs4648014 | 103707899 | 0 | C:C |
|  | 111 | rs4648015 | 103707974 | 0 | C:C |
|  | 112 | rs12645604 | 103708038 | 0 | C:C |
|  | 113 | rs2125212 | 103708319 | 0 | G:G |
|  | 114 | rs230498 | 103708639 | 0.395 | G:A |
|  | 115 | rs4648016 | 103708706 | 0 | C:C |
|  | 116 | rs230499 | 103709019 | 0 | T:T |
|  | 117 | rs4648017 | 103709221 | 0 | T:T |
|  | 118 | rs4648018 | 103709236 | 0 | G:G |
|  | 119 | rs4648019 | 103709301 | 0 | A:A |
|  | 120 | rs4648020 | 103709453 | 0 | A:A |
|  | 121 | rs230500 | 103710760 | 0.395 | G:A |
|  | 122 | rs230539 | 103714570 | 0.411 | A:G |
|  | 123 | rs4648022 | 103715475 | 0 | C:C |
|  | 124 | rs230540 | 103715705 | 0.411 | T:C |
|  | 125 | rs4648023 | 103716092 | 0.012 | A:G |
|  | 126 | rs10023807 | 103716372 | 0 | C:C |
|  | 127 | rs4648024 | 103716605 | 0.058 | C:T |
|  | 128 | rs230541 | 103716823 | 0.465 | A:G |
|  | 129 | rs909332 | 103716913 | 0.056 | A:T |
|  | 130 | rs230542 | 103717493 | 0.407 | C:T |
|  | 131 | rs4648026 | 103718298 | 0 | T:T |
|  | 132 | rs4648028 | 103718966 | 0 | G:G |
|  | 133 | rs4648029 | 103720002 | 0 | C:C |
|  | 134 | rs1801 | 103720092 | 0.411 | G:C |
|  | 135 | rs4648030 | 103720162 | 0 | T:T |
|  | 136 | rs11723120 | 103721589 | 0.4 | G:A |
|  | 137 | rs4699030 | 103722862 | 0.465 | G:C |
|  | 138 | rs4648032 | 103722920 | 0 | A:A |
|  | 139 | rs4648033 | 103722927 | 0 | G:G |
|  | 140 | rs4648034 | 103723297 | 0 | A:A |
|  | 141 | rs1005819 | 103723343 | 0.477 | C:T |
|  | 142 | rs4648036 | 103723419 | 0 | G:G |
|  | 143 | rs4648037 | 103723817 | 0.056 | T:C |
|  | 144 | rs4648038 | 103724227 | 0 | A:A |
|  | 145 | rs4648040 | 103725191 | 0 | C:C |
|  | 146 | rs1610152 | 103725412 | 0.058 | G:C |
|  | 147 | rs1598859 | 103725482 | 0.407 | T:C |
|  | 148 | rs4648041 | 103727142 | 0 | G:G |
|  | 149 | rs4648042 | 103727534 | 0 | T:T |
|  | 150 | rs3774956 | 103727564 | 0.467 | C:T |
|  | 151 | rs4648043 | 103727636 | 0 | C:C |
|  | 152 | rs4648044 | 103727681 | 0.033 | C:T |
|  | 153 | rs3821958 | 103727862 | 0.477 | A:G |
|  | 154 | rs4648046 | 103728068 | 0 | G:G |
|  | 155 | rs4648047 | 103728366 | 0 | T:T |
|  | 156 | rs230546 | 103728988 | 0 | C:C |
|  | 157 | rs1020759 | 103729549 | 0.488 | C:T |
|  | 158 | rs3774959 | 103730152 | 0.407 | G:A |
|  | 159 | rs10489114 | 103730426 | 0 | T:T |
|  | 160 | rs10013190 | 103730810 | 0 | G:G |
|  | 161 | rs4698858 | 103732111 | 0.5 | C:C |
|  | 162 | rs9996972 | 103732939 | 0.056 | A:T |
|  | 163 | rs1020760 | 103733483 | 0.467 | C:G |
|  | 164 | rs4648055 | 103734351 | 0.407 | G:A |
|  | 165 | rs4648058 | 103734629 | 0.474 | G:C |
|  | 166 | rs4698859 | 103734989 | 0 | A:A |
|  | 167 | rs4648063 | 103735735 | 0 | A:A |
|  | 168 | rs4648064 | 103735775 | 0 | A:A |
|  | 169 | rs4648065 | 103736501 | 0 | C:C |
|  | 170 | rs4648068 | 103737343 | 0.407 | A:G |
|  | 171 | rs4648069 | 103737517 | 0 | C:C |
|  | 172 | rs4648072 | 103737738 | 0 | A:A |
|  | 173 | rs4648073 | 103737881 | 0 | G:G |
|  | 174 | rs3774963 | 103738403 | 0.409 | G:C |
|  | 175 | rs4648075 | 103738435 | 0 | G:G |
|  | 176 | rs3774964 | 103738525 | 0.465 | A:G |
|  | 177 | rs4648077 | 103738722 | 0 | A:A |
|  | 178 | rs4648078 | 103738901 | 0 | A:A |
|  | 179 | rs4648081 | 103740285 | 0 | T:T |
|  | 180 | rs4648082 | 103740361 | 0 | A:A |
|  | 181 | rs4648083 | 103740511 | 0 | C:C |
|  | 182 | rs4648084 | 103740625 | 0 | G:G |
|  | 183 | rs4648085 | 103741152 | 0 | C:C |
|  | 184 | rs4648086 | 103741188 | 0.012 | G:A |
|  | 185 | rs4648088 | 103741986 | 0 | G:G |
|  | 186 | rs17032920 | 103742965 | 0 | T:T |
|  | 187 | rs11722146 | 103743667 | 0.4 | G:A |
|  | 188 | rs3774965 | 103743973 | 0.067 | A:C |
|  | 189 | rs12509517 | 103744546 | 0.4 | G:C |
|  | 190 | rs3774966 | 103744874 | 0.022 | T:G |
|  | 191 | rs3755867 | 103745712 | 0.4 | A:G |
|  | 192 | rs4648089 | 103745909 | 0 | T:T |
|  | 193 | rs4648090 | 103746106 | 0.012 | G:A |
|  | 194 | rs4648091 | 103746207 | 0 | T:T |
|  | 195 | rs4648093 | 103746693 | 0 | G:G |
|  | 196 | rs4648094 | 103746807 | 0 | T:T |
|  | 197 | rs4648095 | 103746914 | 0.067 | T:C |
|  | 198 | rs4648096 | 103747055 | 0 | A:A |
|  | 199 | rs4648097 | 103747166 | 0 | G:G |
|  | 200 | rs4648098 | 103747682 | 0 | C:C |
|  | 201 | rs4648099 | 103747855 | 0 | T:T |
|  | 202 | rs4648100 | 103748062 | 0 | G:G |
|  | 203 | rs7682845 | 103748561 | 0 | A:A |
|  | 204 | rs9790601 | 103749001 | 0.389 | A:G |
|  | 205 | rs3774968 | 103750150 | 0.467 | G:A |
|  | 206 | rs4648102 | 103750471 | 0 | C:C |
|  | 207 | rs4648103 | 103750959 | 0 | A:A |
|  | 208 | rs4648104 | 103751029 | 0 | G:G |
|  | 209 | rs4648105 | 103751197 | 0 | G:G |
|  | 210 | rs725200 | 103751344 | 0 | A:A |
|  | 211 | rs4648106 | 103751477 | 0 | G:G |
|  | 212 | rs4648109 | 103752677 | 0 | G:G |
|  | 213 | rs4648110 | 103752867 | 0.081 | T:A |
|  | 214 | rs4648111 | 103752971 | 0 | C:C |
|  | 215 | rs4648112 | 103753056 | 0 | G:G |
|  | 216 | rs4648113 | 103753060 | 0 | C:C |
|  | 217 | rs4648114 | 103753382 | 0 | G:G |
|  | 218 | rs4648115 | 103753449 | 0 | C:C |
|  | 219 | rs4648116 | 103753471 | 0 | G:G |
|  | 220 | rs4648117 | 103753603 | 0.012 | C:T |
|  | 221 | rs3817685 | 103753606 | 0.422 | C:G |
|  | 222 | rs4648118 | 103753739 | 0.012 | G:A |
|  | 223 | rs4648121 | 103754265 | 0 | C:C |
|  | 224 | rs4648122 | 103754305 | 0 | C:C |
|  | 225 | rs4648123 | 103754447 | 0 | C:C |
|  | 226 | rs4648124 | 103754646 | 0 | C:C |
|  | 227 | rs4648125 | 103754811 | 0 | G:G |
|  | 228 | rs4648126 | 103754927 | 0 | A:A |
|  | 229 | rs4648127 | 103754951 | 0.067 | C:T |
|  | 230 | rs4648128 | 103755070 | 0 | A:A |
|  | 231 | rs4648129 | 103755081 | 0 | A:A |
|  | 232 | rs4648130 | 103755084 | 0 | C:C |
|  | 233 | rs230547 | 103755307 | 0.244 | C:T |
|  | 234 | rs4648132 | 103755405 | 0 | A:A |
|  | 235 | rs4648133 | 103755459 | 0.372 | T:C |
|  | 236 | rs4648134 | 103755487 | 0 | G:G |
|  | 237 | rs4648135 | 103755716 | 0.07 | A:G |
|  | 238 | rs4648136 | 103755719 | 0 | C:C |
|  | 239 | rs4648138 | 103755740 | 0 | T:T |
|  | 240 | rs4648139 | 103755864 | 0 | A:A |
|  | 241 | rs4648140 | 103755946 | 0 | C:C |
|  | 242 | rs4648141 | 103755947 | 0.08 | G:A |
|  | 243 | rs1609798 | 103756488 | 0.36 | C:T |
|  | 244 | rs4648142 | 103756493 | 0 | C:C |
|  | 245 | rs4648144 | 103756929 | 0 | C:C |
|  | 246 | rs4648145 | 103757048 | 0 | T:T |
|  | 247 | rs7674640 | 103759828 | 0.45 | C:T |
| NFKB2 | 1 | rs7083038 | 104142775 | 0 | C:C |
|  | 2 | rs1572532 | 104144673 | 0.011 | T:C |
|  | 3 | rs11574845 | 104146373 | 0 | T:T |
|  | 4 | rs7897947 | 104147701 | 0.314 | T:G |
|  | 5 | rs4919632 | 104147717 | 0 | T:T |
|  | 6 | rs11574846 | 104148079 | 0 | G:G |
|  | 7 | rs2295587 | 104148257 | 0 | C:C |
|  | 8 | rs11574847 | 104149051 | 0 | C:C |
|  | 9 | rs4919633 | 104149186 | 0 | G:G |
|  | 10 | rs11574849 | 104149686 | 0 | G:G |
|  | 11 | rs4919634 | 104150424 | 0 | G:G |
|  | 12 | rs11574851 | 104150949 | 0.058 | C:T |
|  | 13 | rs11574852 | 104151465 | 0 | A:A |
|  | 14 | rs1056890 | 104152760 | 0.186 | G:A |
| RELA | 1 | rs1466462 | 65175940 | 0.244 | G:C |
|  | 2 | rs10896027 | 65177336 | 0.244 | C:G |
|  | 3 | rs1049728 | 65177693 | 0 | G:G |
|  | 4 | rs7119750 | 65179167 | 0.389 | C:T |
|  | 5 | rs11227247 | 65179429 | 0.384 | A:C |
|  | 6 | rs1144792 | 65179658 | 0 | A:A |
|  | 7 | rs12721572 | 65179923 | 0 | T:T |
|  | 8 | rs1108922 | 65180506 | 0 | C:C |
|  | 9 | rs11606329 | 65182840 | 0 | A:A |
|  | 10 | rs11568298 | 65182895 | 0 | T:T |
|  | 11 | rs11568297 | 65183569 | 0 | A:A |
|  | 12 | rs2306365 | 65183922 | 0.384 | G:A |
|  | 13 | rs732072 | 65184144 | 0.035 | G:A |
|  | 14 | rs11557248 | 65185782 | 0 | C:C |
|  | 15 | rs11820062 | 65186512 | 0.372 | C:T |
|  | 16 | rs11607323 | 65187543 | 0 | C:C |
|  | 17 | rs7101916 | 65187936 | 0.395 | C:T |
| RELB | 1 | rs11667466 | 50195183 | 0 | A:A |
|  | 2 | rs7251460 | 50198612 | 0.071 | A:T |
|  | 3 | rs7255042 | 50199015 | 0 | T:T |
|  | 4 | rs10423543 | 50200112 | 0.023 | A:C |
|  | 5 | rs2060251 | 50200864 | 0.07 | A:C |
|  | 6 | rs874743 | 50205076 | 0.117 | G:A |
|  | 7 | rs35891370 | 50210940 | 0.172 | A:G |
|  | 8 | rs7257048 | 50212125 | 0 | A:A |
|  | 9 | rs35912475 | 50214572 | 0.235 | C:T |
|  | 10 | rs4803789 | 50215209 | 0.254 | G:T |
|  | 11 | rs4803790 | 50215299 | 0.164 | A:G |
|  | 12 | rs4803791 | 50215423 | 0.401 | G:A |
|  | 13 | rs2288918 | 50220639 | 0.449 | T:C |
|  | 14 | rs6509177 | 50221846 | 0.441 | A:G |
|  | 15 | rs12609547 | 50223849 | 0.452 | G:T |
|  | 16 | rs10424046 | 50227876 | 0.467 | G:C |
|  | 17 | rs2376869 | 50232820 | 0 | T:T |
|  | 18 | rs1560725 | 50235627 | 0.467 | C:T |
| REL | 1 | rs2568372 | 60962477 | 0 | G:G |
|  | 2 | rs12477512 | 60965738 | 0.023 | G:A |
|  | 3 | rs6545835 | 60966056 | 0 | G:G |
|  | 4 | rs6719147 | 60967295 | 0 | A:A |
|  | 5 | rs10208155 | 60968543 | 0 | T:T |
|  | 6 | rs13422089 | 60969745 | 0 | C:C |
|  | 7 | rs842648 | 60969985 | 0.151 | A:G |
|  | 8 | rs13022703 | 60971026 | 0 | G:G |
|  | 9 | rs12713428 | 60971617 | 0 | A:A |
|  | 10 | rs13429481 | 60971710 | 0 | G:G |
|  | 11 | rs10185028 | 60972243 | 0 | A:A |
|  | 12 | rs842647 | 60972975 | 0.163 | G:A |
|  | 13 | rs10171095 | 60974637 | 0 | G:G |
|  | 14 | rs842644 | 60974996 | 0 | A:A |
|  | 15 | rs6729789 | 60975074 | 0 | A:A |
|  | 16 | rs6545836 | 60976477 | 0 | G:G |
|  | 17 | rs34695944 | 60978354 | 0.012 | T:C |
|  | 18 | rs10193964 | 60980168 | 0 | A:A |
|  | 19 | rs6707682 | 60981772 | 0 | A:A |
|  | 20 | rs6708517 | 60982457 | 0 | G:G |
|  | 21 | rs9752570 | 60985533 | 0 | T:T |
|  | 22 | rs7604989 | 60987297 | 0 | A:A |
|  | 23 | rs859779 | 60987916 | 0 | T:T |
|  | 24 | rs13031237 | 60989633 | 0.012 | G:T |
|  | 25 | rs842616 | 60990002 | 0.174 | A:C |
|  | 26 | rs10172912 | 60993546 | 0 | A:A |
|  | 27 | rs9309331 | 60995119 | 0 | G:G |
|  | 28 | rs842619 | 60999843 | 0.174 | A:G |
|  | 29 | rs1429265 | 61006586 | 0 | T:T |

*Genetic variation data for the NFKB family genes was obtained from the HapMap project (www.hapmap.org) for 137 Chinese Han Beijing (CHB) population. MAF indicates minor allele frequency.
