# Supplementary material for: Identification of haplotype tag single nucleotide polymorphisms within the nuclear factor-κB family genes and their clinical relevance in patients with major trauma
Source: Crit Care. 2015 Mar 20;19(1):95. doi: 10.1186/s13054-015-0836-6 (PMC4404128; doi:10.1186/s13054-015-0836-6)
Supplement: Additional file 2: Table S2. — Single nucleotide polymorphisms with a minor allele frequency ≥0.05 in the NF-κB-family genes in the Chinese Han population. [file 13054_2015_836_MOESM2_ESM.docx]

Table S2 SNPs with MAF ≥ 0.05 in the NFKB family Genes in Chinese Han Population*

| Gene | No. | rs Number | Position | MAF | Alleles | Location on the Gene | Region |
| --- | --- | --- | --- | --- | --- | --- | --- |
| NFKB1 | 1 | rs3774932 | 103643223 | 0.489 | G:A | 1708 | Intron1 |
|  | 2 | rs3774933 | 103645369 | 0.419 | T:C | 3854 | Intron1 |
|  | 3 | rs3774934 | 103646506 | 0.322 | G:A | 4991 | Intron1 |
|  | 4 | rs17032705 | 103652004 | 0.43 | G:A | 10489 | Intron1 |
|  | 5 | rs3774936 | 103653167 | 0.378 | A:T | 11652 | Intron1 |
|  | 6 | rs3774937 | 103653283 | 0.372 | T:C | 11768 | Intron1 |
|  | 7 | rs3774938 | 103657469 | 0.433 | A:G | 15954 | Intron1 |
|  | 8 | rs4235404 | 103659095 | 0.354 | C:G | 17580 | Intron1 |
|  | 9 | rs1599961 | 103662599 | 0.43 | G:A | 21084 | Intron1 |
|  | 10 | rs1585215 | 103663504 | 0.378 | T:C | 21989 | Intron1 |
|  | 11 | rs1585214 | 103663563 | 0.477 | C:T | 22048 | Intron1 |
|  | 12 | rs1585213 | 103663728 | 0.43 | C:T | 22213 | Intron1 |
|  | 13 | rs1598856 | 103665145 | 0.488 | G:A | 23630 | Intron1 |
|  | 14 | rs230535 | 103667612 | 0.395 | C:A | 26097 | Intron2 |
|  | 15 | rs170731 | 103667933 | 0.4 | A:T | 26418 | Intron2 |
|  | 16 | rs230534 | 103668071 | 0.381 | C:T | 26556 | Intron2 |
|  | 17 | rs230533 | 103669113 | 0.4 | G:A | 27598 | Intron2 |
|  | 18 | rs230532 | 103669197 | 0.395 | A:T | 27682 | Intron2 |
|  | 19 | rs230531 | 103669407 | 0.395 | A:G | 27892 | Intron2 |
|  | 20 | rs230530 | 103673010 | 0.465 | A:G | 31495 | Intron3 |
|  | 21 | rs4647992 | 103674377 | 0.07 | C:T | 32862 | Intron3 |
|  | 22 | rs230529 | 103676448 | 0.453 | C:T | 34933 | Intron4 |
|  | 23 | rs230528 | 103676615 | 0.453 | T:G | 35100 | Intron4 |
|  | 24 | rs230526 | 103677855 | 0.464 | G:A | 36340 | Intron4 |
|  | 25 | rs230525 | 103677907 | 0.395 | A:G | 36392 | Intron4 |
|  | 26 | rs4648004 | 103680136 | 0.081 | A:G | 38621 | Intron5 |
|  | 27 | rs4648006 | 103680588 | 0.058 | C:T | 39073 | Intron5 |
|  | 28 | rs118882 | 103682036 | 0.405 | C:T | 40521 | Intron5 |
|  | 29 | rs230521 | 103682357 | 0.453 | G:C | 40842 | Intron5 |
|  | 30 | rs230520 | 103684641 | 0.395 | A:G | 43126 | Intron5 |
|  | 31 | rs230519 | 103685778 | 0.395 | C:T | 44263 | Intron5 |
|  | 32 | rs93059 | 103687547 | 0.452 | G:A | 46032 | Intron5 |
|  | 33 | rs230516 | 103690280 | 0.4 | A:G | 48765 | Intron5 |
|  | 34 | rs230515 | 103690463 | 0.379 | A:G | 48948 | Intron 5 |
|  | 35 | rs230514 | 103690968 | 0.395 | A:G | 49453 | Intron5 |
|  | 36 | rs4648011 | 103694479 | 0.453 | T:G | 52964 | Intron5 |
|  | 37 | rs230510 | 103695201 | 0.467 | T:A | 53686 | Intron5 |
|  | 38 | rs230509 | 103697300 | 0.4 | A:G | 55785 | Intron5 |
|  | 39 | rs13117745 | 103697738 | 0.081 | C:T | 56223 | Intron5 |
|  | 40 | rs230505 | 103700386 | 0.453 | T:G | 58871 | Intron5 |
|  | 41 | rs230504 | 103700596 | 0.395 | C:T | 59081 | Intron5 |
|  | 42 | rs230491 | 103703651 | 0.4 | C:T | 62136 | Intron5 |
|  | 43 | rs230492 | 103704817 | 0.398 | G:A | 63302 | Intron5 |
|  | 44 | rs230493 | 103705253 | 0.395 | T:A | 63738 | Intron5 |
|  | 45 | rs1598861 | 103705733 | 0.163 | A:C | 64218 | Intron5 |
|  | 46 | rs230495 | 103706336 | 0.465 | G:A | 64821 | Intron5 |
|  | 47 | rs2293970 | 103707018 | 0.057 | A:T | 65503 | Intron5 |
|  | 48 | rs230496 | 103707527 | 0.476 | A:G | 66012 | Intron6 |
|  | 49 | rs230498 | 103708639 | 0.395 | G:A | 67124 | Intron6 |
|  | 50 | rs230500 | 103710760 | 0.395 | G:A | 69245 | Intron6 |
|  | 51 | rs230539 | 103714570 | 0.411 | A:G | 73055 | Intron6 |
|  | 52 | rs230540 | 103715705 | 0.411 | T:C | 74190 | Intron6 |
|  | 53 | rs4648024 | 103716605 | 0.058 | C:T | 75090 | Intron6 |
|  | 54 | rs230541 | 103716823 | 0.465 | A:G | 75308 | Intron6 |
|  | 55 | rs909332 | 103716913 | 0.056 | A:T | 75398 | Intron6 |
|  | 56 | rs230542 | 103717493 | 0.407 | C:T | 75978 | Intron7 |
|  | 57 | rs1801 | 103720092 | 0.411 | G:C | 78577 | Intron8 |
|  | 58 | rs11723120 | 103721589 | 0.4 | G:A | 80074 | Intron9 |
|  | 59 | rs4699030 | 103722862 | 0.465 | G:C | 81347 | Intron9 |
|  | 60 | rs1005819 | 103723343 | 0.477 | C:T | 81828 | Intron10 |
|  | 61 | rs4648037 | 103723817 | 0.056 | T:C | 82302 | Intron10 |
|  | 62 | rs1610152 | 103725412 | 0.058 | G:C | 83897 | Intron11 |
|  | 63 | rs1598859 | 103725482 | 0.407 | T:C | 83967 | Intron11 |
|  | 64 | rs3774956 | 103727564 | 0.467 | C:T | 86049 | Intron11 |
|  | 65 | rs3821958 | 103727862 | 0.477 | A:G | 86347 | Intron11 |
|  | 66 | rs1020759 | 103729549 | 0.488 | C:T | 88034 | Intron 11 |
|  | 67 | rs3774959 | 103730152 | 0.407 | G:A | 88637 | Intron11 |
|  | 68 | rs4698858 | 103732111 | 0.5 | C:C | 90596 | Intron 11 |
|  | 69 | rs9996972 | 103732939 | 0.056 | A:T | 91424 | Intron11 |
|  | 70 | rs1020760 | 103733483 | 0.467 | C:G | 91968 | Intron11 |
|  | 71 | rs4648055 | 103734351 | 0.407 | G:A | 92836 | Intron12 |
|  | 72 | rs4648058 | 103734629 | 0.474 | G:C | 93114 | Intron12 |
|  | 73 | rs4648068 | 103737343 | 0.407 | A:G | 95828 | Intron14 |
|  | 74 | rs3774963 | 103738403 | 0.409 | G:C | 96888 | Intron15 |
|  | 75 | rs3774964 | 103738525 | 0.465 | A:G | 97010 | Intron15 |
|  | 76 | rs11722146 | 103743667 | 0.4 | G:A | 102152 | Intron16 |
|  | 77 | rs3774965 | 103743973 | 0.067 | A:C | 102458 | Intron16 |
|  | 78 | rs12509517 | 103744546 | 0.4 | G:C | 103031 | Intron16 |
|  | 79 | rs3755867 | 103745712 | 0.4 | A:G | 104197 | Intron16 |
|  | 80 | rs4648095 | 103746914 | 0.067 | T:C | 105399 | Intron17 |
|  | 81 | rs9790601 | 103749001 | 0.389 | A:G | 107486 | Intron19 |
|  | 82 | rs3774968 | 103750150 | 0.467 | G:A | 108635 | Intron19 |
|  | 83 | rs4648110 | 103752867 | 0.081 | T:A | 111352 | Intron22 |
|  | 84 | rs3817685 | 103753606 | 0.422 | C:G | 112091 | Intron22 |
|  | 85 | rs4648127 | 103754951 | 0.067 | C:T | 113436 | Intron23 |
|  | 86 | rs230547 | 103755307 | 0.244 | C:T | 113792 | Intron23 |
|  | 87 | rs4648133 | 103755459 | 0.372 | T:C | 113944 | Intron23 |
|  | 88 | rs4648135 | 103755716 | 0.07 | A:G | 114201 | Intron23 |
|  | 89 | rs4648141 | 103755947 | 0.08 | G:A | 114432 | Intron23 |
|  | 90 | rs1609798 | 103756488 | 0.36 | C:T | 114973 | Intron23 |
|  | 91 | rs7674640 | 103759828 | 0.45 | C:T | 118313 | 3'flanking |
| NFKB2 | 92 | rs7897947 | 104147701 | 0.314 | T:G | 3845 | intron9 |
|  | 93 | rs11574851 | 104150949 | 0.058 | C:T | 7093 | exon19（Asn→Asn） |
|  | 94 | rs1056890 | 104152760 | 0.186 | G:A | 8904 | 3'flanking |
| RELA | 95 | rs1466462 | 65175940 | 0.244 | G:C | 11080 | 3'flanking |
|  | 96 | rs10896027 | 65177336 | 0.244 | C:G | 9684 | 3'flanking |
|  | 97 | rs7119750 | 65179167 | 0.389 | C:T | 7853 | intron10 |
|  | 98 | rs11227247 | 65179429 | 0.384 | A:C | 7591 | intron10 |
|  | 99 | rs2306365 | 65183922 | 0.384 | G:A | 3098 | intron5 |
|  | 100 | rs11820062 | 65186512 | 0.372 | C:T | 508 | intron1 |
|  | 101 | rs7101916 | 65187936 | 0.395 | C:T | -916 | 5'flanking |
| RELB | 102 | rs7251460 | 50198612 | 0.071 | A:T | 2066 | intron2 |
|  | 103 | rs2060251 | 50200864 | 0.07 | A:C | 4318 | intron2 |
|  | 104 | rs874743 | 50205076 | 0.117 | G:A | 8530 | intron3 |
|  | 105 | rs35891370 | 50210940 | 0.172 | A:G | 14394 | intron4 |
|  | 106 | rs35912475 | 50214572 | 0.235 | C:T | 18026 | intron4 |
|  | 107 | rs4803789 | 50215209 | 0.254 | G:T | 18663 | intron4 |
|  | 108 | rs4803790 | 50215299 | 0.164 | A:G | 18753 | intron4 |
|  | 109 | rs4803791 | 50215423 | 0.401 | G:A | 18877 | intron4 |
|  | 110 | rs2288918 | 50220639 | 0.449 | T:C | 24093 | intron6 |
|  | 111 | rs6509177 | 50221846 | 0.441 | A:G | 25300 | intron7 |
|  | 112 | rs12609547 | 50223849 | 0.452 | G:T | 27303 | intron7 |
|  | 113 | rs10424046 | 50227876 | 0.467 | G:C | 31330 | intron9 |
|  | 114 | rs1560725 | 50235627 | 0.467 | C:T | 39081 | 3'flanking |
| REL | 115 | rs842648 | 60969985 | 0.151 | A:G | 7773 | intron1 |
|  | 116 | rs842647 | 60972975 | 0.163 | G:A | 10763 | intron2 |
|  | 117 | rs842616 | 60990002 | 0.174 | A:C | 27790 | intron4 |
|  | 118 | rs842619 | 60999843 | 0.174 | A:G | 37631 | intron7 |

*Genetic variation data for the NFKB family gene was obtained from the HapMap project (www.hapmap.org) for 137 Chinese Han Beijing (CHB) population. MAF indicates minor allele frequency.
