# Supplementary figures and images for: Identification of haplotype tag single nucleotide polymorphisms within the nuclear factor-κB family genes and their clinical relevance in patients with major trauma
Source: Crit Care. 2015 Mar 20;19(1):95. doi: 10.1186/s13054-015-0836-6 (PMC4404128; doi:10.1186/s13054-015-0836-6)

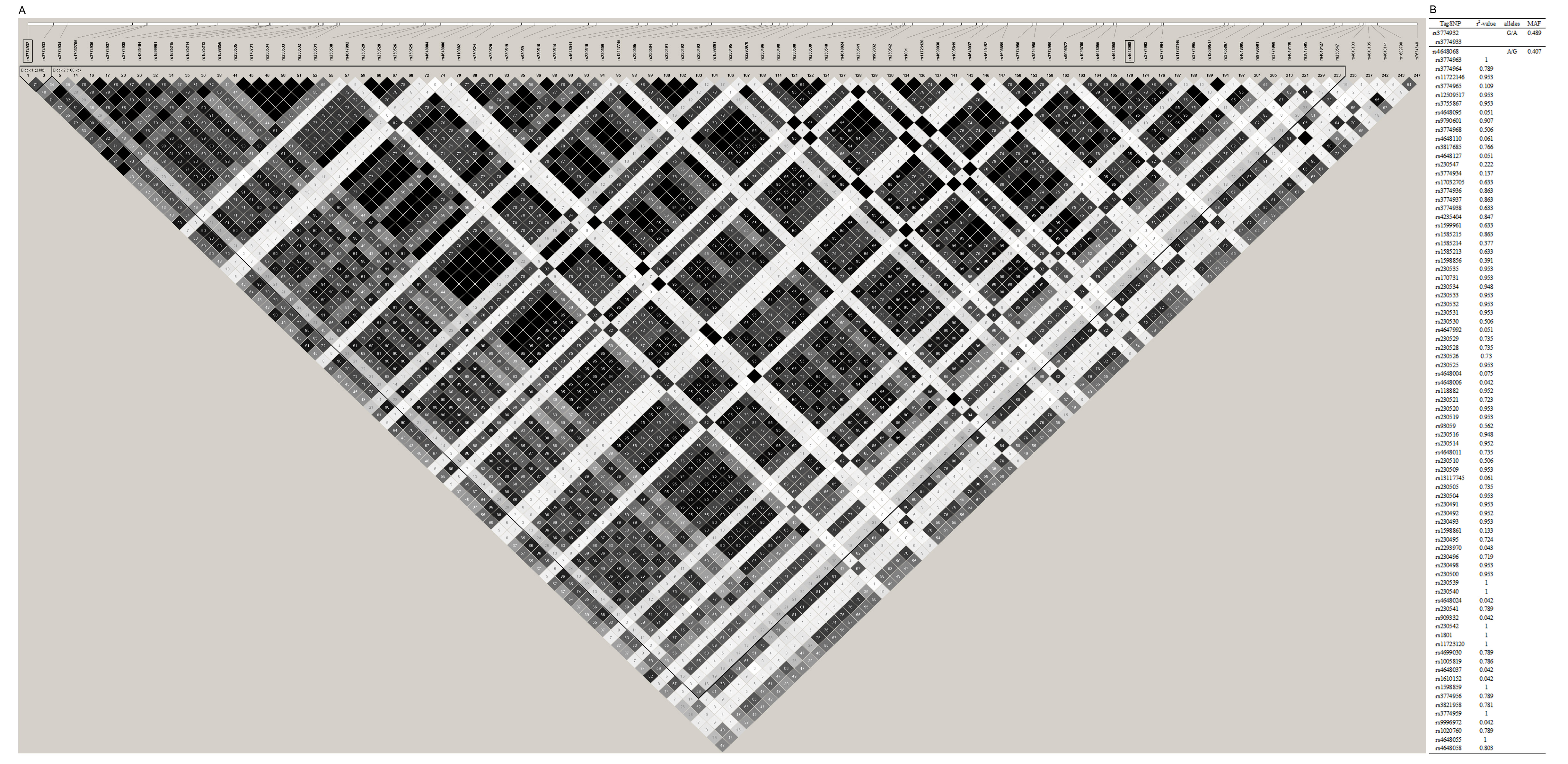

Supplement: Additional file 3: Figure S1. — Overview of selected haplotype tag single nucleotide polymorphisms (SNPs) within the entire NFKB1 gene and their characteristics. (A) Location of the 91 SNPs within the NFKB1 gene and 5-kb up- and downstream regions with a minor allele frequency ≥5%. The selected two haplotype tag (ht)SNPs are indicated by boxes. Linkage disequilibrium (LD) plot of the 91 SNPs in the 126.0 kb region is displayed by using r 2-black and white color scheme. Black represents very high LD (r 2 = 1), and white indicates the absence of correlation (r 2 = 0) between SNPs. (B) The two htSNPs and SNPs that are indirectly measured by them are listed with corresponding r 2 values. Major and minor alleles of the selected tag SNPs are given with their frequencies, on the basis of the HapMap data for the Chinese individuals from Beijing. [file 13054_2015_836_MOESM3_ESM.jpeg]
